# Assessment of Four Molecular Markers as Potential DNA Barcodes for Red Algae *Kappaphycus* Doty and *Eucheuma* J. Agardh (Solieriaceae, Rhodophyta)

**DOI:** 10.1371/journal.pone.0052905

**Published:** 2012-12-20

**Authors:** Ji Tan, Phaik-Eem Lim, Siew-Moi Phang, Dang Diem Hong, H. Sunarpi, Anicia Q. Hurtado

**Affiliations:** 1 Institute of Biological Sciences, University of Malaya, Kuala Lumpur, Malaysia; 2 Institute of Ocean and Earth Sciences (IOES), University of Malaya, Kuala Lumpur, Malaysia; 3 Institute of Biotechnology, Vietnamese Academy of Science and Technology, Cau Giay, Hanoi, Vietnam; 4 Faculty of Science and Mathematics, Mataram University, Mataram, Lombok, Indonesia; 5 Integrated Services for the Development of Aquaculture and Fisheries, Tabuc Suba, Iliolo City, Philippines; Aberystwyth University, United Kingdom

## Abstract

DNA barcoding has been a major advancement in the field of taxonomy, seeing much effort put into the barcoding of wide taxa of organisms, macro and microalgae included. The mitochondrial-encoded *cox*1 and plastid-encoded *rbc*L has been proposed as potential DNA barcodes for rhodophytes, but are yet to be tested on the commercially important carrageenophytes *Kappaphycus* and *Eucheuma*. This study gauges the effectiveness of four markers, namely the mitochondrial *cox*1, *cox*2, *cox*2-3 spacer and the plastid *rbc*L in DNA barcoding on selected *Kappaphycus* and *Eucheuma* from Southeast Asia. Marker assessments were performed using established distance and tree-based identification criteria from earlier studies. Barcoding patterns on a larger scale were simulated by empirically testing on the commonly used *cox*2-3 spacer. The phylogeny of these rhodophytes was also briefly described. In this study, the *cox*2 marker which satisfies the prerequisites of DNA barcodes was found to exhibit moderately high interspecific divergences with no intraspecific variations, thus a promising marker for the DNA barcoding of *Kappaphycus* and *Eucheuma*. However, the already extensively used *cox*2-3 spacer was deemed to be in overall more appropriate as a DNA barcode for these two genera. On a wider scale, c*ox*1 and *rbc*L were still better DNA barcodes across the rhodophyte taxa when practicality and cost-efficiency were taken into account. The phylogeny of *Kappaphycus* and *Eucheuma* were generally similar to those earlier reported. Still, the application of DNA barcoding has demonstrated our relatively poor taxonomic comprehension of these seaweeds, thus suggesting more in-depth efforts in taxonomic restructuring as well as establishment.

## Introduction

The introduction of DNA barcodes by Herbert and co-workers [1], [2], [3] has brought about large impacts on the advancement of systematic biology; where short, easily amplified regions of DNA exhibiting large variation among species, yet sufficiently variable within species, are constantly used for species delineation, identification as well as archiving with reference to known, established species [4], [5], [6]. The Barcode of Life Data System (BOLD) is notably the largest initiative in establishing a worldwide DNA barcode library, signifying its importance and popularity for the scientific community [7], [8], [9]. The usefulness of DNA barcoding is evident when dealing with taxa displaying phenotypic plasticity throughout diphasic or triphasic life cycles as well as taxa involving cryptic species. These phenomena are generally predominant in marine macroalgae, thereby enticing the application of DNA barcoding, as reported in numerous studies encompassing the order Gelidiales [10], Gigartinales, [11], [12], [13], Graciliariales [14], [15], Laminariales [16], and Fucales [17]. Studies on DNA barcoding over broader taxa of rhodophytes have also been reported with promising results [18], [19], [20].

The rhodophytes *Kappaphycus* and *Eucheuma*, commercially known as “cottonii” and “spinosum”, respectively are widely established as lucrative sources of carrageenan, with Indonesia and the Philippines being the largest carrageenophyte producers worldwide [21]. Despite being extensively farmed, the morphologically diverse nature of *Kappaphycus* and *Eucheuma* still poses difficulties in species identification [22], [23], [24], [25], [26], [27], [28], even leading to the cultivation of mixed populations that inevitably reduces overall yield [29]. These have resulted in the subsequent employment of molecular phylogenetic studies which all share one main objective – to infer and understand the phylogenetic relationships between *Kappaphycus* and *Eucheuma* congeners. As of now, various molecular markers have been introduced for the molecular taxonomy of these carrageenophytes, namely the mitochondrial-encoded partial *cox*1 and *cox*2-3 spacer, nuclear-encoded ribosomal Internal Transcribed Spacer (ITS) and 28S large subunit (LSU), plastid-encoded *rbc*L, RuBisCO spacer and the 23S Universal Plastid Amplicon (UPA) [23], [28], [30], [31]. However, the suitability of these genetic markers as DNA barcodes are to date, unassessed.

Having high quality DNA barcodes for *Kappaphycus* and *Eucheuma* would hasten the identification and recording of these seaweeds throughout the globe, particularly those within Southeast Asian waters, where it is believed that many species are yet to be described. Accurate identification of undescribed species would be most useful in developing improved commercial varieties, preferably ones with higher carrageenan yield, faster growth as well as better disease resistance. Apart from species diversity, data on the distribution of *Kappaphycus* and *Eucheuma* would also be useful in the detection of invasive strains [23], [32]. The potential benefits of DNA barcoding for *Kappaphycus* and *Eucheuma* underline the main purpose of the present study, which is to gauge the efficacy of four molecular markers, including the commonly used mitochondrial *cox*1 and plastid *rbc*L barcodes, the mitochondrial *cox*2-3 spacer and a newly designed *cox*2 molecular marker as potential DNA barcodes for selected *Kappaphycus* and *Eucheuma* specimens found in Southeast Asia. Marker assessment and comparison were carried out using the tree-based DNA identification technique as well as the distance technique- *Best Match* (BM), *Best Close Match* (BCM) and *All Species Barcodes* (ASB) criteria [1], [33]. Additionally, the effect of larger datasets on the accuracy and robustness of DNA barcoding was assessed by incorporating sequences of the widely used *cox*2-3 spacer marker which includes specimens collected from different parts of the world, to that generated in the present study.

## Materials and Methods

### Ethics Statement

*Kappaphycus* and *Eucheuma* specimens were not collected from any national parks or protected areas, thus not requiring any specific permits for sampling. Specimens were collected from open coastal areas as well as from aquaculture sites with consents from the respective owners. Members of the two genera are not endangered or protected species.

### Sample Processing

Details of samples used in this study are summarized in Table 1. Specimens were either sampled directly from farms, retrieved via snorkeling or scuba diving. Sample preservation and genomic DNA extraction were based on protocols described by [29]. Details on molecular markers and primers utilized in this study are summarized in Table 2. The mitochondrial encoded cytochrome *c* oxidase subunit 2 (*cox*2) primers were designed based on the *cox*2 region of the complete mitochondrial genome of *Chondrus crispus* (GenBank no. NC_001677) [34]. PCR parameters of the *cox*2 molecular marker are similar to that of *rbc*L, but with 30 amplification cycles. Amplicons were electrophoresed using a SYBR®Safe (Invitrogen, USA) stained 1.0% agarose gel, prior to purification using LaboPass^TM^ (Cosmo Gentech, Korea) gel and PCR purification kits. Purified products were sent to Lucigen (Taiwan) for ABI SoLiD sequencing.

**Table 1:** Details of samples used in this study.

No.	Sample Name	Operational Taxonomic Unit (OTU)	Sampling Location	Collection Code	GenBank Accession Numbers			
					*Cox*1	*Cox*2-3 spacer	*Cox*2	*rbc*L
1	*Kappaphycus alvarezii* 13 “*Buaya*” *	KA1	Sabangkat, Sabah, Malaysia	PSM11996-UMSS0144	-	JN663762	-	-
2	*Kappaphycus alvarezii* 18 “*Tambalang Giant*” *		Sabangkat, Sabah, Malaysia	PSM12001-UMSS0154	-	JN663768	-	-
3	*Kappaphycus alvarezii* 52 “*Buaya*” *		Omadal, Sabah, Malaysia	PSM12029-UMSS0196	-	JN663763	-	-
4	*Kappaphycus alvarezii* 53 “*Tangan-tangan*” *		Omadal, Sabah, Malaysia	PSM12030-UMSS0198	-	JN663773	-	-
5	*Kappaphycus alvarezii* 58 “*Tangan-tangan*” *		Omadal, Sabah, Malaysia	PSM12035-UMSS0203	JX624014	JN663774	JX624043	JX623985
6	*Kappaphycus alvarezii* 63 “*Tambalang Giant*”		Sisipan, Sabah, Malaysia	PSM12043-UMSS0214		JN663769		-
7	*Kappaphycus alvarezii* 89 “*Tambalang* Brown” *		Sandakan, Sabah, Malaysia	PSM12059-UMSS0230	JX624015	JN663766	JX624044	JX623986
8	*Kappaphycus alvarezii* 103*		Sabangkat, Sabah, Malaysia	PSM12072-UMSS0243	JX624016	JN663776	JX624045	JX623987
9	*Kappaphycus alvarezii* 109 “*Tangan-tangan*”		Semporna, Sabah, Malaysia	PSM12078-UMSS0249	-	JN663775	-	-
10	*Kappaphycus alvarezii* 121 “*Tambalang* Green” *		Pangkor Island, Perak, Malaysia	PSM12105-UMSS0260	JX624017	JN663772	JX624046	JX623988
11	*Kappaphycus alvarezii* 123 “*Tambalang* Brown” *		Pangkor Island, Perak, Malaysia	PSM12107-UMSS0262	-	JN663767	-	-
12	*Kappaphycus alvarezii* BA*		Semporna, Sabah, Malaysia	-	-	JN234760	-	-
13	*Kappaphycus alvarezii* BN*		Semporna, Sabah, Malaysia	-	-	JN234759	-	-
14	*Kappaphycus alvarezii* YF*		Semporna, Sabah, Malaysia	-	-	JN234762	-	-
15	*Kappaphycus alvarezii* 433		Teluk Ekas, Indonesia	PSM12290-UMSS0433	JX624018	JX624072	JX624047	JX623989
16	*Kappaphycus alvarezii* ZAM4 “*Milo*”		Zamboanga City, Mindanao, Philippines	AQHZAM004-UMSS0380	JX624019	JX624073	JX624048	JX623990
17	*Kappaphycus alvarezii* V7 “Dark Green”		Son Hai, Vietnam	PSM12380-UMSS0525	JX624020	JX624074	JX624049	JX623991
18	*Kappaphycus alvarezii* E3*		Venezuela	-	-	AY687427	-	-
19	*Kappaphycus alvarezii* 2614*		Hawaii	-	-	FJ554862	-	-
20	*Kappaphycus alvarezii* UR13*		Tanzania	-	-	JQ713902	-	-
21	*Kappaphycus alvarezii* E130*	KA2	Tanzania	-	-	AY687427	-	-
22	*Kappaphycus alvarezii* Reef4*		Paje-Jambiani, Tanzania	-	-	JQ713901	-	-
23	*Kappaphycus alvarezii* E16*		Madagascar	-	-	AY687430	-	-
24	*Kappaphycus alvarezii* E57*	KA3	Hawaii	-	-	AY687432	-	-
25	*Kappaphycus alvarezii* E71*		Hawaii	-	-	AY687433	-	-
26	*Kappaphycus alvarezii* 919*		Hawaii	-	-	FJ554860	-	-
27	*Kappaphycus alvarezii* 3955*		Hawaii	-	-	FJ554861	-	-
28	*Kappaphycus striatum* 1 “Yellow Flower” *	KS1	Sabangkat, Sabah, Malaysia	PSM11984-UMSS0128	JX624021	JN663779	JX624050	JX623992
29	*Kappaphycus striatum* 31 “Green Flower” *		Sabangkat, Sabah, Malaysia	PSM12011-UMSS0170	JX624022	JN663780	JX624051	JX623993
30	*Kappaphycus striatum* 59 “Green Flower” *		Bum-Bum Island, Malaysia	PSM12039-UMSS0208	JX624023	JN663777	JX624052	JX623994
31	*Kappaphycus striatum* 60 “Green Flower” *		Bum-Bum Island, Malaysia	PSM12040-UMSS0209	-	JN663778	-	-
32	*Kappaphycus striatum* AG*		Semporna, Sabah, Malaysia	-	-	JN234763	-	-
33	*Kappaphycus striatum* GF*		Semporna, Sabah, Malaysia	-	-	JN234765	-	-
34	*Kappaphycus striatum* GTF*		Semporna, Sabah, Malaysia	-	-	JN234764	-	-
35	*Kappaphycus striatum* 460		Kertasari, Indonesia	PSM12293-UMSS0460	JX624024	JX624075	JX624053	JX623995
36	*Kappaphycus striatum* GUI4 “*Cottonii*”		Guimaras Is. Panay, Philippines	AQHGUI004-UMSS0360	JX624025	JX624076	JX624054	JX623996
37	*Kappaphycus striatum* SIT5 “*Cottonii* light green (sacol)”		Sitangkai, Tawi Mindanao, Philippines	AQHSIT005-UMSS0394	JX624026	JX624077	JX624055	JX623997
38	*Kappaphycus striatum* V6 “*Payaka* Green”		Cam Ranh, Vietnam	PSM12379-UMSS0524	JX624027	JX624078	JX624056	JX623998
39	*Kappaphycus striatum* E89*		Philippines	-	-	AY687434	-	-
40	*Kappaphycus striatum* 83*	KS2	Sabangkat, Sabah, Malaysia	PSM12053-UMSS0224	JX624028	JN663781	JX624057	JX623999
41	*Kappaphycus striatum* 98*		Sabangkat, Sabah, Malaysia	PSM12067-UMSS0238	JX624029	JN663782	JX624058	JX624000
42	*Kappaphycus striatum* 105*		Sabangkat, Sabah, Malaysia	PSM12074-UMSS0245	JX624030	JN663783	JX624059	JX624001
43	*Kappaphycus striatum* D13*		Semporna, Sabah, Malaysia	-	-	JN645177	-	-
44	*Kappaphycus striatum* D14*		Semporna, Sabah, Malaysia	-	-	JN645178	-	-
45	*Kappaphycus striatum* SIT4 “*Kab-kab* green”		Sitangkai, Tawi Mindanao, Philippines	AQHSIT004-UMSS0393	JX624031	JX624079	JX624060	JX624002
46	*Kappaphycus striatum* E117*		Indonesia	-	-	AY687435	-	-
47	*Kappaphycus striatum* E48*		Indonesia	-	-	AY687431	-	-
48	*Kappaphycus* sp. 14 “*Aring-aring*” *	KAr	Sabangkat, Sabah, Malaysia	PSM11997-UMSS0146	-	JN663784	-	-
49	*Kappaphycus* sp. 49 “*Aring-aring*” *		Sabangkat, Sabah, Malaysia	PSM12026-UMSS0192	JX624032	JN663785	JX624061	JX624003
50	*Kappaphycus* sp. 93 “*Aring-aring*” *		Sabangkat, Sabah, Malaysia	PSM12063-UMSS0234	JX624033	JN663786	JX624062	JX624004
51	*Kappaphycus* sp. 115 “*Aring-aring*”		Sabangkat, Sabah, Malaysia	PSM12100-UMSS0255	JX624034	JX624080	JX624063	JX624005
52	*Eucheuma denticulatum* 44 “*Spinosum*” *	ED1	Sabangkat, Sabah, Malaysia	PSM12021-UMSS0187	JX624035	JN663787	JX624064	JX624006
53	*Eucheuma denticulatum* 45 “*Spinosum*” *		Sabangkat, Sabah, Malaysia	PSM12022-UMSS0188	JX624036	JN663788	JX624065	JX624007
54	*Eucheuma denticulatum* 46 “*Spinosum*” *		Sabangkat, Sabah, Malaysia	PSM12023-UMSS0189	-	JN663789	-	-
55	*Eucheuma denticulatum* 56 “*Spinosum*” *		Omadal, Sabah, Malaysia	PSM12033-UMSS0201	JX624037	JN663790	JX624066	JX624008
56	*Eucheuma denticulatum* 57 “*Spinosum*” *		Omadal, Sabah, Malaysia	PSM12034-UMSS0202	-	JN663791	-	-
57	*Eucheuma denticulatum* 99 “*Spinosum*” *		Sabangkat, Sabah, Malaysia	PSM12068-UMSS0239	-	JN663792	-	-
58	*Eucheuma denticulatum* DM*		Semporna, Sabah, Malaysia	-	-	JN234756	-	-
59	*Eucheuma denticulatum* AD*		Semporna, Sabah, Malaysia	-	-	JN980403	-	-
60	*Eucheuma denticulatum* AB*		Semporna, Sabah, Malaysia	-	-	JN234758	-	-
61	*Eucheuma denticulatum* E13*		Indonesia	-	-	AY687429	-	-
62	*Eucheuma denticulatum* 454	ED2	Kertasari, Indonesia	PSM12292-UMSS0454	JX624038	JX624081	JX624067	JX624009
63	*Eucheuma denticulatum* BOH5 “*Spinosum*”		Bohol, Central Visayas, Philippines	AQHBOH005-UMSS0371	JX624039	JX624082	JX624068	JX624010
64	*Eucheuma denticulatum* 41 “*Cacing*”		Sabangkat, Sabah, Malaysia	PSM12018-UMSS0181	JX624040	JX624083	JX624069	JX624011
65	*Eucheuma denticulatum* 42 “*Cacing*”		Sabangkat, Sabah, Malaysia	PSM12019-UMSS0183	JX624041	JX624084	JX624070	JX624012
66	*Eucheuma denticulatum* 97 “*Cacing*”		Sabangkat, Sabah, Malaysia	PSM12066-UMSS0237	JX624042	JX624085	JX624071	JX624013
67	*Eucheuma denticulatum* CG *		Semporna, Sabah, Malaysia	-	-	JN234757	-	-
68	*Eucheuma denticulatum* E32*		Indonesia	-	-	AY687437	-	-
69	*Eucheuma denticulatum* 888*		Hawaii	-	-	FJ554859	-	-
70	*Eucheuma denticulatum* E8*	ED3	Madagascar	-	-	AY687428	-	-
71	*Eucheuma denticulatum* PAC5*		Paje-Jambiani	-	-	JQ713903	-	-
72	*Eucheuma denticulatum* E60*		Mauritius	-	-	AY687439	-	-
73	*Eucheuma denticulatum* 3953		Hawaii	-	-	FJ561733	-	-
74	*Eucheuma platycladum* E65*	EP	Tanzania	-	-	AY687423	-	-
75	*Eucheuma platycladum* E111*		Kenya	-	-	AY687422	-	-
76	*Solieria* 120	-	Merambong, Johor, Malaysia	PSM12104-UMSS0259	-	JN663793	-	-

Specimens were grouped into Operational Taxonomic Units (OTU) for selected analyses. Dashes (−) indicate non-available or irrelevant data. Asterisks (*) indicate samples where corresponding *cox*2-3 spacer sequences were obtained from the GenBank and used for *Large Dataset Assessment*.

**Table 2:** Primer details and corresponding annealing temperatures for the *cox*1, *cox*2, *cox*2-3 spacer and *rbc*L genetic markers used in this study.

DNA Markers	Primers	Primer Sequences*	Reference	Annealing temperature, T_m_
*Cox*1	COXI43F C622F C880R COXI1549R	5′-TCAACAAATCATAAAGATATTGGWACT-3′ 5′-CCTGTNTTAGCAGGWGCTATTACAATGC-3′ 5′-ACAGTATACATATGATGNGCTCAAAC-3′ 5′-AGGCATTTCTTCAAANGTATGATA-3′	[46], [47]	52°C
*Cox*2-3 spacer	*Cox*2_for *Cox*3_rev	5′-GTACCWTCTTTDRGRRKDAAATGTGATGC-3′ 5′-GGATCTACWAGATGRAAWGGATGTC-3′	[55]	50°C
*Cox*2	*Kcox*2_F71 *Kcox*2_R671	5′-TTCAAGATCCTGCAACTCC-3′ 5′-ATTTCACTGCATTGGCCAT-3′	This Study	51°C
*rbc*L	F-7 F-577 R-753 R-rbcS start	5′-AACTCTGTAGTAGAACGNACAAG-3′ 5′-GTATATGAAGGTCTAAAAGGTGG-3′ 5′-GCTCTTTCATACATATCTTCC-3′ 5′-GTTCTTTGTGTTAATCTCAC-3′	[58], [59]	50°C

Ambiguous nucleotide codes are in accordance to IUPAC: K = G/T; R =  A/G; W =  A/T; N =  A/T/C/G.

Resulting electropherograms were viewed, truncated at terminals and contigs generated using ChromasPro V1.5 (Technelysium Pty Ltd). Multiple sequence alignments were computed using ClustalX V2.0 [35] and converted into NEXUS format prior to subsequent analyses.

Considering the poor taxonomic status of *Kappaphycus* and *Eucheuma*, certain molecular marker assessments were conducted using two different criteria, where (i) species were named and processed based on Operational Taxonomic Units (OTU) (Table 1) [36], or (ii) original species names were used with no alterations whatsoever throughout the analyses, herein termed non-OTU. The 2^nd^ criterion is only applicable under the *Large Dataset Assessment*. An OTU in this context refers to a cluster of species-specific specimens constituting a monophyletic clade which is (i) sufficiently variable genetically from the sister taxa such that bifurcating patterns are observed or (ii) geographically distinct. Morphological criteria were not applied due to the extensive morphological plasticity of these red algae.

### Distance-based Assessment

Distance analyses were performed using TaxonDNA's Species Identifier v1.7.7 [33] for each molecular marker dataset. The pairwise distances for intra- and interspecific frequencies; calculated using both the uncorrected and the Kimura 2-parameter (K2P) corrected pairwise distances, were plotted to observe overlaps in genetic variability, if present. These pairwise distances were computed using the (i) total overlap range and (ii) 90% overlapping range- the largest 5% of the intraspecific and lowest 5% of the interspecific samples excluded. Minimum base pairs in common for distance calculations were set at 300 bp for all molecular markers assessed. The effectiveness of respective molecular markers was tested based on the *Best Match* (BM) and *Best Close Match* (BCM) criteria comprehensively described by Meier and co-workers [33]. In short, the BM criterion assigns a species name to the query sequence based on its best barcode match, regardless of the magnitude of similarity between the query and the barcode sequences. BCM, on the other hand involves the initial identification of the best-matched barcode, determination of whether the barcode is sufficiently similar to the query, followed lastly by the assignment of a species name [33]. The smallest interspecific distance generated using the Pairwise Summary module for each molecular marker was used as threshold value for BM and BCM computations.

### Tree-based Assessment

Neighbor-Joining (NJ) trees were generated based on the Kimura 2-parameter model using default PAUP 4.0b10 [37] settings for each DNA marker to provide visual displays of genetic variation within and between species. Tree nodal supports were generated via 1,000 bootstrap replicates. Resulting NJ trees were analyzed and processed using Figtree v1.3.1 [38]. For all trees, the success of species identification was determined based on the criteria proposed in [1] and [33]. Under Hebert and co-workers [1], identification was considered a success when the query clusters along with all conspecific sequences; and considered a failure when the query matches conspecific sequences occurring in multiple clusters or clades within the tree. Ambiguous were all singletons within the dataset. Meier and co-workers [33] used a revised, and more stringent identification criteria for the tree-based approach. Queries were considered correctly identified when in polytomy with conspecifics, or at least one node into a clade of conspecifics. Misidentification were those queries in polytomy with only allospecific sequences or those at least one node into an allospecific clade. Queries without conspecific sequences or queries sister to conspecifics were considered as ambiguous or unidentified. Additionally, the phylogeny of *Kappaphycus* and *Eucheuma* were interpreted and briefly described based on the resulting NJ, Maximum Likelihood (ML), Maximum Parsimony (MP) and Bayesian Inference (BI) trees.

### Large Dataset Assessment

The *cox*2-3 spacer, currently the most widely used genetic marker for *Kappaphycus* and *Eucheuma*, were used to simulate empirically the effectiveness of a molecular marker in relatively larger datasets. *Cox*2-3 spacer sequences from recent studies [23], [29], [32], [39] were obtained from the GenBank. Specimens of ambiguous or uncertain identity were excluded from the dataset. Multiple sequence alignments were generated and subjected to similar K2P distance and tree-based analyses. Datasets were analyzed based on the application of the OTU and non-OTU criteria. An additional distance-based identification criterion termed *All Species Barcodes* (ASB) was also employed using Species Identifier v1.7.7 [33]. This relatively more subtle approach compares the query to barcodes generated using the same threshold for BM and BCM. Queries were considered as successfully identified when matched with at least two conspecific barcodes of the species in question. Ambiguous were all queries followed by only one conspecific barcode or only a portion of the conspecific sequences. Queries were designated misidentified when matched with allospecific barcodes.

Phylogenetic trees were also inferred for the *cox*2-3 spacer based on Maximum Likelihood (ML), Maximum Parsimony (MP) and Bayesian (BI) algorithms. *Solieria* was used as the outgroup for each analyses [29]. Parsimony analyses were conducted using PAUP 4.0b10 [37] as heuristic searches using 1,000 bootstrapping replications; with 100 stepwise random sequence addition and tree bisection reconnection (TBR) branch swapping. All characters were assigned unordered and unweighted. Retention indices (RI) and Consistency indices (CI) were also generated.

ML analysis was performed using raxmlGUI [40] and BI via Mr. Bayes v3.2.1 [41], [42]. Best fit nucleotide substitution models were determined for each DNA region using Kakusan 4 [43], generating command files for RAxML and input files for MrBayes. ML trees were inferred using the ML + thorough bootstrap algorithm, with the GTR+GAMMA model over 20 independent searches and 1,000 non-parametric bootstrap replicates. Identical sequences were not omitted for the analysis. For the BI analyses, two sets of four Monte Carlo Markov Chains (MCMC) were performed in parallel over 2,000,000 generations, with trees sampled every 500^th^ generation. Tracer v1.5 (http://196 tree.bio.ed.ac.uk/software/tracer/) was used to assess convergence of log likelihood values, where 100,000 generations were discarded as burn-ins, well after stationarity was achieved. Results were used to generate a 50% majority-rule consensus tree. All trees were again analysed and processed using Figtree v1.3.1 [38].

## Results

All 29 sequences were continuous and easily aligned for all four molecular markers, generating datasets of different final lengths: *cox*1 (1,411 bp), *cox*2 (575 bp), *cox*2-3 spacer (365 bp) and for *rbc*L (1,464bp). *Cox*1 exhibited the most phylogenetically informative sites of 277 (19.6%) characters, followed by *rbc*L with 126 (8.6%) characters, *cox*2 with 115 (20%) characters and the *cox*2-3 spacer with 76 (20.8%) characters. All sequences were deposited into GenBank (see Table 1). The *cox*1 sequences amplified in this study encompasses the ones by Saunders et al. [19] and Robba et al. [18].

### Distance-based Assessment

**Figure 1:**
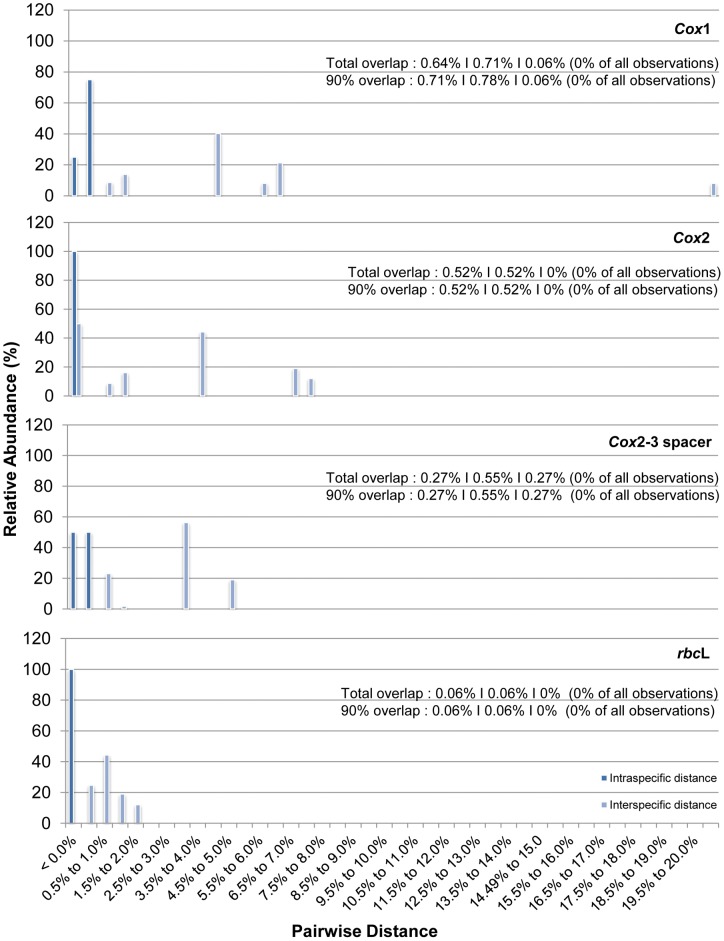
Plot of intra- and interspecific genetic distances for the *cox*1, *cox*2, *cox*2-3 spacer and *rbc*L DNA markers. Numeric values are arranged according to: the difference between the smallest interspecific but intrageneric distance and the largest intraspecific distance|smallest pairwise distance between interspecific but intrageneic sequences|largest pairwise distance between intraspecific sequences; followed by the number of observations affected (in brackets).

Pairwise distances (based on the corrected Kimura 2-parameter) showing the intra- and interspecific genetic variability for *cox*1, *cox*2, *cox*2-3 spacer and *rbc*L were plotted in Figure 1. Both the corrected and uncorrected pairwise distances generated similar results for *cox*2, *cox*2-3 spacer and *rbc*L. No overlapping in terms of intra- and interspecific genetic divergence was observed for all four molecular markers. However, the *rbc*L marker showed the least distance (0.06% differences for total and 90% overlaps) between the smallest pairwise distance among interspecific but intrageneric samples and the largest pairwise distance among intraspecific sequences- smaller “barcoding gap”. This was followed by *cox*2-3 spacer (0.06% for total and 90% overlaps), *cox*2 (0.52% for total and 90% overlaps) and *cox*1 (K2P: 0.64% for total overlaps and 0.71% for 90% overlaps; uncorrected pairwise distance: 0.63% for total overlaps and 0.70% for 90% overlaps).

Results based on the *Best Match* (BM) and *Best Close Match* (BCM) criteria are as follows: All four genetic markers were able to correctly identify all 29 species based on the BM criteria. For the BCM criteria, *rbc*L showed the highest success in identification with 100%, followed by *cox*2 (96.6%), *cox*2-3 spacer (93.1%) and *cox*1 (79.3%). No matches were recorded at values 3.44%, 6.89% and 20.68% for *cox*2, *cox*2-3 spacer and *cox*1 respectively.

### Tree-based Assessment

**Figure 2:**
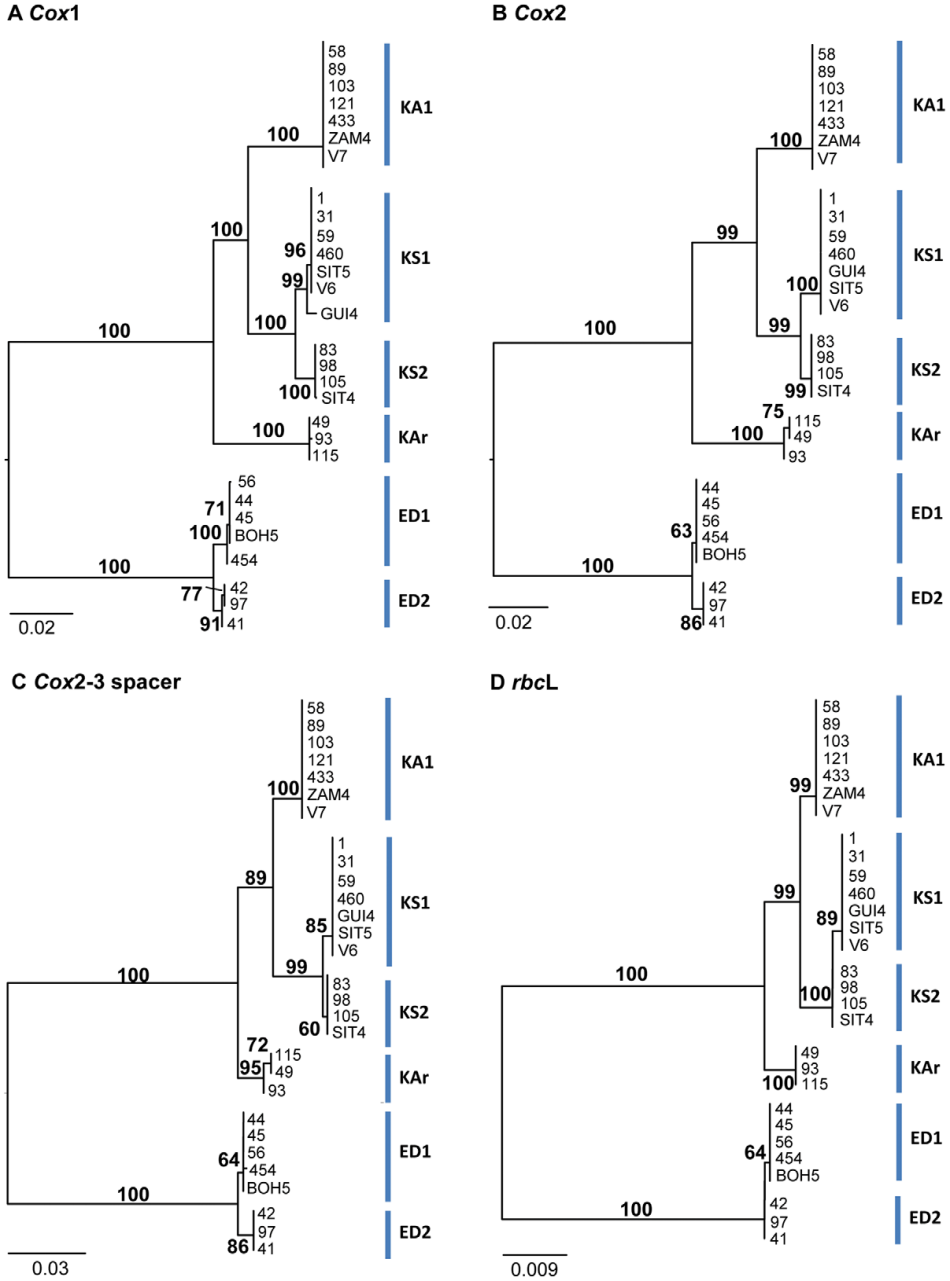
Neighbour Joining (**NJ**) **trees of selected** ***Kappaphycus*** **and** ***Eucheuma*** **from Southeast Asia based on** (**a**) ***cox*** **1;** (**b**) ***cox*** **2;** (**c**) ***cox*** **2-3 spacer;** (**d**) ***rbc*** **L molecular markers.** Numbers at node indicate corresponding bootstrap values over 1,000 replicates. Clade annotations represent Operational Taxonomic Units (OTU), where KA  =  *Kappaphycus alvarezii*; KS =  *Kappaphycus striatum*; KAr  =  *Kappaphycus* sp. “*Aring-aring*”; ED =  *Eucheuma denticulatum*.

Resulting NJ phylogenetic trees are shown in Figure 2. Similar tree topologies were observed based on the *cox*1, *cox*2, *cox*2-3 spacer and the *rbc*L molecular markers, all showing an apparent phylogenetic delineation between *Kappaphycus* and *Eucheuma* i.e. Neighbor Joining bootstrap supports (NJ-BS) of 100% respectively. Better resolution was observed for the mitochondrial-encoded genetic markers compared to the plastid-encoded *rbc*L counterpart on the species level, with relatively higher discrimination in the clustering of specimens.

Tree-based DNA identification using Hebert et al.'s [1] criteria showed 100% identification success across all species for all four genetic markers. Apart from the plastid *rbc*L DNA marker, identification success saw a decrease with the application of Meier et al.'s [33] revised identification criteria. For the *cox*1 marker, identification success was recorded at 89.7%, with the remaining 10.3% sequences assigned as ambiguous. Both the *cox*2 and *cox*2-3 spacer showed identification success of 96.6%, with 3.4% ambiguity.

Tree topologies based on ML, MP and BI analyses were identical to that of the NJ trees and were not shown (to be distributed upon request). For the monophyletic *Kappaphycus* clade, four subclades were generated, denoted as OTUs KA1, KS1, KS2 and KAr; corresponding to *K. alvarezii*, *K. striatum* 1, *K. striatum* 2 and *Kappaphycus* sp. “*Aring-aring*” respectively. All *K. alvarezii* specimens were inferred to be the same species with no significant genetic variation by all four markers (NJ-BS = 99–100%). Two genotypic strains of *K. striatum*-KS1 (1, 31, 59, 460, GUI4, SIT5 and V6) and KS2 (83, 98, 105 and SIT4) were observed with high support (NJ-BS = 99–100%) for each marker, although the clustering was less obvious for *rbc*L. *Cox*1 demonstrated more specific delineation within species, where the Philippine GUI4 specimen was inferred to be genetically different from the rest of the KS1 specimens (NJ-BS = 99%). Based on all four markers, *Kappaphycus* sp. “*Aring-aring*”, first reported by Tan and co-workers (2012), formed a highly supported monophyletic clade (NJ-BS = 95–100%) sister to that of *K. alvarezii* and *K. striatum* (NJ-BS = 100%). Specimens 49 and 115, which were both from Sabah, were shown with moderate support (NJ-BS = 72% and 75%) to be more closely related to one another compared to that of sample 93 by the *cox*2-3 spacer and *cox*2 genetic marker respectively.

*Eucheuma denticulatum* specimens formed a monophyletic clade composed of subclades termed as ED1 (44, 45, 56, 454 and BOH5) and ED2 (41, 42 and 97) for all four molecular markers (NJ-BS = 100%). Similar to that observed for *K. striatum*, the assortment of ED1 and ED2 was not that discrete for the *rbc*L marker. Conversely, the *cox*1 marker exhibited relatively better intraspecific delineation, where the Indonesian 454 specimen was shown to be different from the remaining ED1 samples (NJ-BS = 100%). Although moderately supported (NJ-BS = 77%), samples 42 and 97 were inferred by *cox*1 to be distinct from that of sample 41.

Despite the equal ability of all four markers to cluster species consistently and somewhat accurately, the *cox*1, *cox*2 and *rbc*L NJ trees showed increased robustness in terms of nodal supports, of which the *cox*1 marker exhibited more specific resolution in terms of intraspecific genetic variations.

### Large Dataset Assessment

Multiple sequence alignments with a length of 341bp were generated from the dataset incorporating all selected 76 *cox*2-3 spacer sequences (including the *Solieria* outgroup), representing ten OTUs or five non-OTUs. *Cox*2-3 spacer sequences generated from this study were truncated to allow better comparisons with shorter GenBank counterparts. 118 phylogenetically informative and 192 constant characters were recorded.

**Figure 3:**
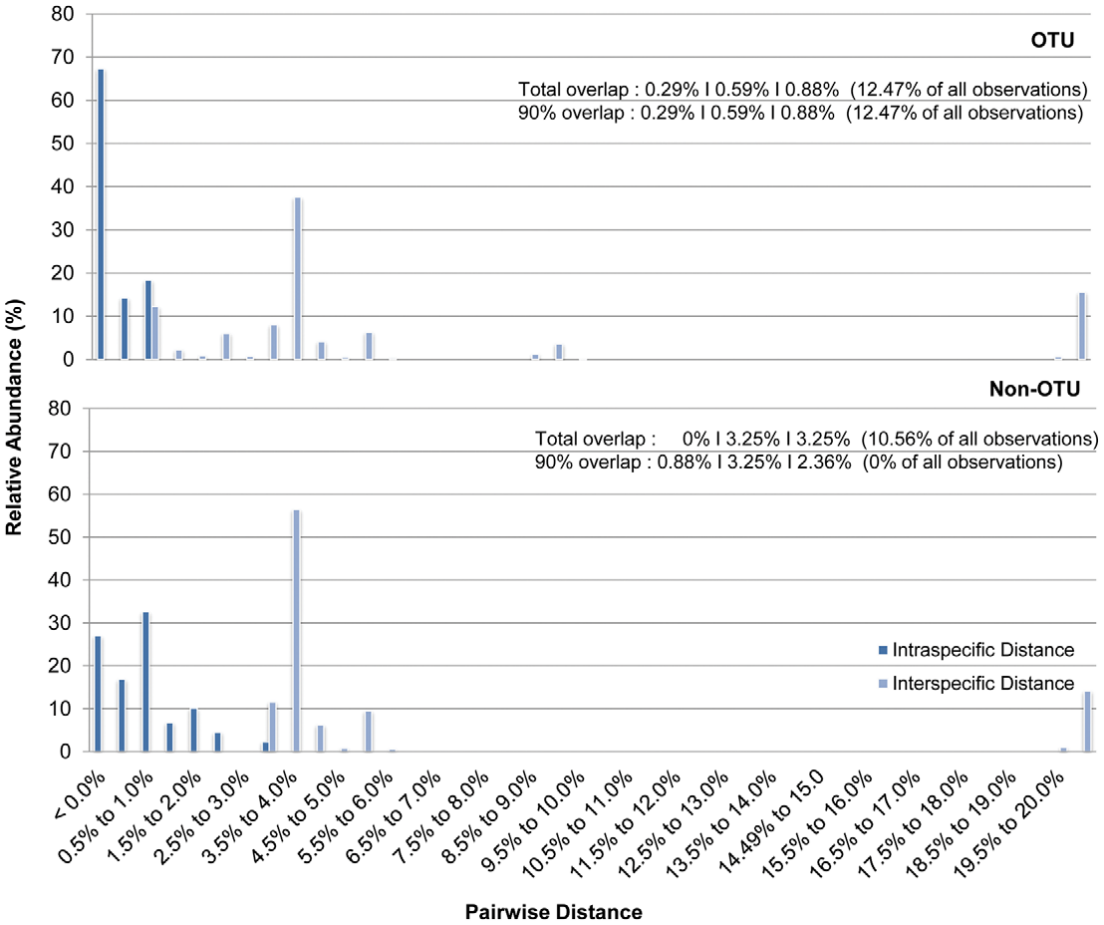
Plot of intra- and interspecific genetic distances of the *cox*2-3 spacer with the application of OTU and non-OTU criteria. Numeric values are arranged according to: the difference between the smallest interspecific but intrageneric distance and the largest intraspecific distance|smallest pairwise distance between interspecific but intrageneic sequences|largest pairwise distance between intraspecific sequences; followed by the number of observations affected (in brackets).

Figure 3 illustrates the intraspecific and interspecific genetic divergence based on the K2P model between OTUs and non-OTUs. For the former, a pairwise distance overlap of 0.29% was observed between the smallest interspecific and the largest intraspecific sequences; whereas no overlaps were observed for the latter.

**Figure 4:**
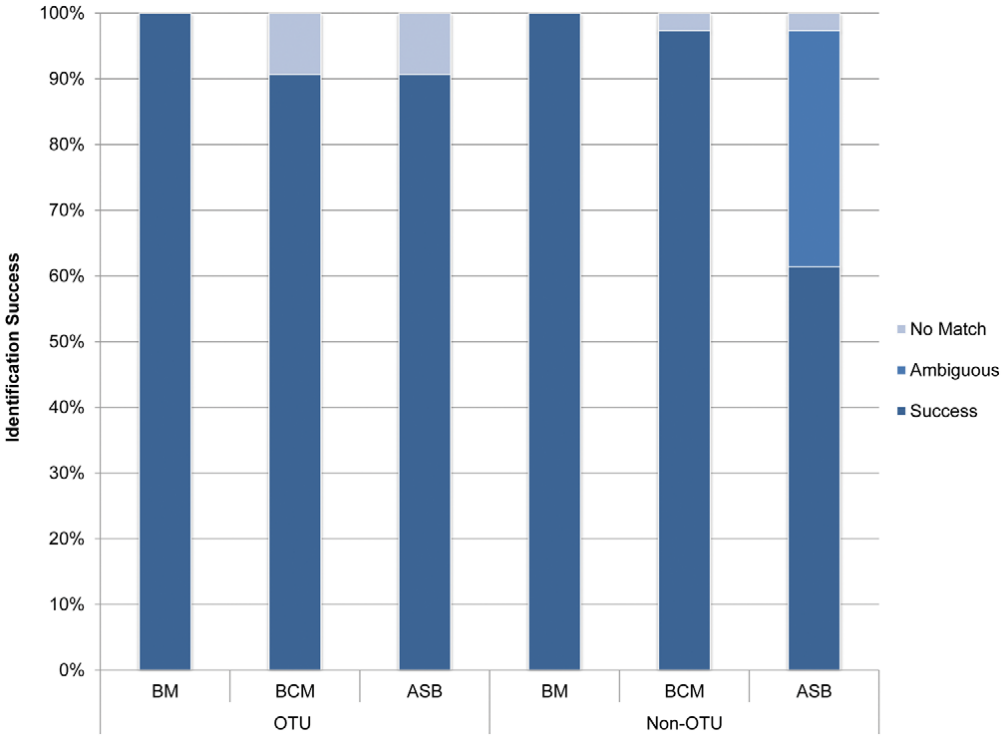
Identification success of the *cox*2-3 spacer. *Kappaphycus* and *Eucheuma* samples were categorized under either Operational Taxonomic Units (OTU) or non-OTU conditions. Results were generated based on the *Best Match* (BM), *Best Close Match* (BCM) and *All Species Barcodes* (ASB) criteria.

Identification successes based on the BM and BCM criteria are shown in Figure 4. All species were correctly identified under BM for OTU and non-OTU analyses. Under BCM selection, 90.66% of samples were correctly identified when species were considered as OTUs, and 97.33% when species were not considered as OTUs. No matches were found for the remaining queries. Under the *All Species Barcodes* (ASB) identification criteria, an identification success of 90.66% and 61.33% were observed for OTU and non-OTU species categorization respectively. 36% of sequences were considered as ambiguous under the non-OTU criteria whereas the remaining queries were designated matchless.

**Figure 5:**
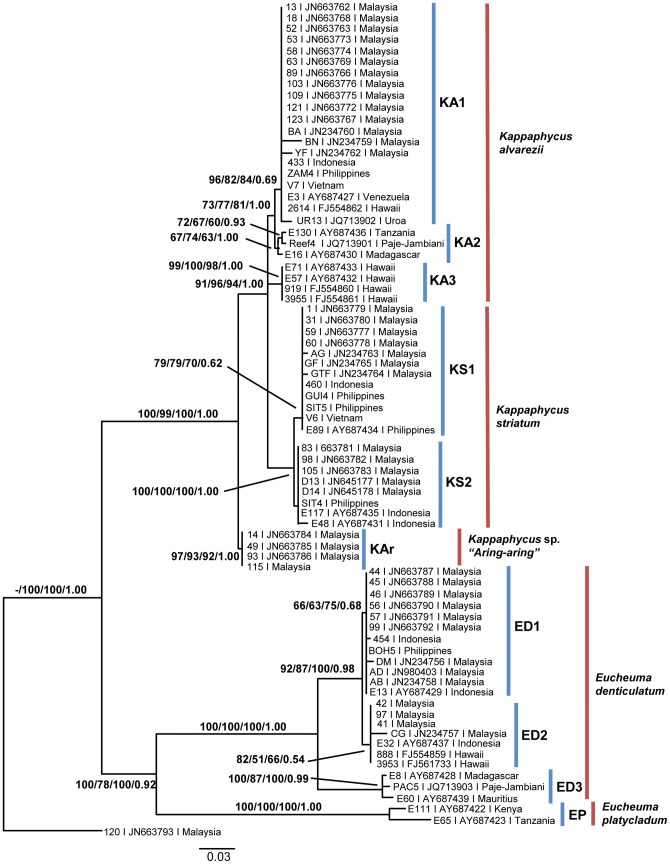
Maximum likelihood 50% majority-rule consensus tree based on the *cox*2-3 spacer. Number at nodes is arranged according to NJ bootstrap support/ML bootstrap support/MP bootstrap support/Bayesian posterior probabilities. *Large dataset assessment*: blue lines indicate Operational Taxonomic Units (OTU), whereas red lines represent non-OTU clusters. The *Solieria* outgroup was excluded in the NJ analysis.

Due to the similarity in tree topologies based on the NJ, ML, MP and BI algorithms, and for simplicity, resulting data are compiled and depicted as Figure 5. The NJ, MP and BI phylogenetic trees are provided as supporting data Figure S1, S2 and S3 respectively. Tree-based DNA identification is assessed using the NJ tree without taking into account the *Solieria* outgroup not used in the NJ dataset. In accordance to identification criteria by Hebert and co-workers [1], 100% identification success is reported when tree-based identification is based on the OTU concept. When taxa are queried using conventional taxonomic naming (non-OTU), percentage of successful identification is reduced to 94.6%, where the remaining 5.4% indicated misidentifications. Application of Meier et. al.'s [33] identification criteria returned comparatively lower successful identification rates, where 95.9% success and 4.1% ambiguity was recorded for OTUs; and 67.6% successful identification, 27% ambiguity and 5.4% misidentification for non-OTUs.

Phylogenetic analyses of the entire *cox*2-3 spacer dataset is inclusive of the *Solieria* outgroup. A total of 32 most parsimonious trees were produced by Parsimony analysis, with a Retention Index (RI) and Consistency Index (CI) of 0.9748 and 0.7934 respectively. The tree topology is generally similar to the NJ trees generated for molecular marker assessment, with most specimens clustering into existing clades. Remaining GenBank samples, mostly of different species or locality, were inferred as additional, discrete monophyletic clades. Nevertheless, the overall tree topology is still congruent to that of phylogenetic trees reported in earlier studies [23], [28], [29] and will not be discussed in detail. Nodal supports of the phylogenetic tree (Figure 5) were arranged in the order of Neighbor Joining bootstrap supports (NJ-BS), Maximum Likelihood bootstrap supports (ML-BS), Maximum Parsimony bootstrap supports (MP-BS) and Bayesian Inference posterior probabilities (BI-PP). Major nodes were mostly moderate to highly supported despite the addition in taxa. Fairly high support were observed for the monophyly of *Kappaphycus* (NJ-BS = 100%; ML-BS = 99%; MP-BS = 100%; BI-PP = 1.00) and *Eucheuma* (NJ-BS = 100%; ML-BS = 78%; MP-BS = 100%; BI-PP = 0.92) used in this study. Although strongly supported (NJ-BS = 91%; ML-BS = 96%; MP-BS = 94%; BI-PP = 1.00), polytomy is observed between *K. alvarezii* (KA1, KA2 and KA3) and *K. striatum*; where KA2 (NJ-BS = 67%; ML-BS = 74%; MP-BS = 63%; BI-PP = 1.00) and KA3 (NJ-BS = 99%; ML-BS = 100%; MP-BS = 98%; BI-PP = 1.00) represent *K. alvarezii* specimens from the African and Hawaiian regions respectively. *Kappaphycus* sp. “*Aring-aring*” (NJ-BS = 97%; ML-BS = 93%; MP-BS = 92%; BI-PP = 1.00) and *Eucheuma denticulatum* (NJ-BS = 100%; ML-BS = 100%; MP-BS = 100%; BI-PP = 1.00) were inferred to be monophyletic as well. Clade EP (NJ-BS = 100%; ML-BS = 100%; MP-BS = 100%; BI-PP = 1.00) comprises of *Eucheuma platycladum* collected from around Southeast Africa.

## Discussion

The dataset in the present study represents a small conglomerate of selected and commonly available *Kappaphycus* and *Eucheuma* samples from Southeast Asia. Sampling size is restricted to an amount supposedly cost-effective for molecular marker assessments as potential DNA barcodes. Owing to the relatively scarce records of *Kappaphycus* and *Eucheuma* in the GenBank, this study will also serve as a preliminary work to increase the amount of reference data using the potential DNA barcode, which can eventually be established as a barcode library.

### Molecular Marker Assessment

The accuracy of a DNA barcode is largely determined by the magnitude of overlap between intraspecific variation and interspecific divergence. Ideally, the absence of an overlap would render species identification straightforward but this condition is virtually non-existent in very large datasets, in which the less the overlap, the more accurate it is for species identification [3], [6], [44]. Results indicated that no overlaps were observed for the *cox*1, *cox*2, *cox*2-3 spacer and the *rbc*L genetic markers and have minimal impact on the accuracy of species identification for *Kappaphycus* and *Eucheuma* in this study (Figure 1). However, the absence of overlaps may be attributed to the small taxa limited to Southeast Asia (particularly so the genus *Eucheuma*) at this time, and is expected to change as more samples of different species or from different geographical locations are included in the future [6], [45]. The presence of a “barcoding gap” (the absence of overlaps between intra- and interspecific genetic variations) may also be explained by the relatively lower genetic diversity amongst red algae as compared to those observed in arthropods.

All four molecular markers collectively showed an identification success of 100% for the BM criteria. Under the BCM criteria, the plastid encoded *rbc*L gene exhibited the highest identification success of 100% whereas the mitochondrial encoded *cox*2, *cox*2-3 spacer and *cox*1 spacer recorded slightly lower success of 96.6%, 93.1% and 79.3% respectively. Misidentification of species was not reported for all four molecular markers. The eventual increase in conspecific DNA sequences for *Kappaphycus* and *Eucheuma* is expected to reduce the probability of queries not meeting any matching sequences. All these results reflect the relatively less variable *rbc*L region as compared to mitochondrial counterparts, which was also reported by Geraldino et al. [46] and Yang et al. [47]. Although widely championed as a good potential DNA barcode, the relatively higher intraspecific variation of the *cox*1 marker (<0.43% for *Kappaphycus*; <0.07% for *Eucheuma*) requires caution in avoiding misidentifications. Similar or higher intraspecific patterns were pointed out in previous studies associated with rhodophytes [18], [19], [46], [47], [48], [49].

Tree-based DNA identification approach returned 100% success in species identification for all four molecular markers using the criteria by Hebert and co-workers [1]. Although the application of the relatively stricter identification criteria by Meier and co-workers [33] generally lowered the successful identification scores, they were still higher compared to the results derived from the distance-based approach. For instance, *cox*1 and *cox*2 showed a higher identification success of 89.7% and 96.6% respectively. Ambiguous identification in this study was mostly caused by queries that formed a sister group to a cluster of conspecific sequences. This is also expected to decrease as more reference sequences are deposited in the GenBank for *Kappaphycus* and *Eucheuma*. Contrary to the better results obtained by the tree-based method in this study, empirical studies involving much larger taxa coverage have reported the preference of distance-based assessment over tree-based ones in terms of accuracy and robustness [33], [50].

Based on both the distance and tree-based DNA identification approaches, the relatively conserved nature and the absence of overlaps between inter- and intraspecific genetic variability of the *rbc*L gene serves as a better potential DNA barcode for *Kappaphycus* and *Eucheuma*. However, the reduced genetic variation would also imply the incapability of the *rbc*L marker to detect incipient speciation or genetic diversity within species [5], [51]. This will not only result in an underestimate of the actual genetic richness of these seaweeds, but will most likely overestimate interspecific variation as well due to the unavailability of closely related species [6]. The drawbacks of the plastid encoded *rbc*L marker can be accounted for using supplementary molecular data generated from the relatively more variable, mitochondrial derived *cox*1, *cox*2 or *cox*2-3 spacer. The concept of combined molecular data, in this case of molecular markers, is not new considering the occasional failures of the DNA barcodes in correctly and consistently identifying species, as observed in Karner blue butterflies [52], seagrasses [53] and flowering plants [54]. Utilization of genomic DNA from different origin i.e. mitochondrial, plastid or nuclear with different evolutionary rates would offer a better picture with respect to gene genealogy and evolutionary lineage.

The *cox*1 and *cox*2 DNA markers hold better potential as supplementary DNA barcodes compared to the *cox*2-3 spacer because of their protein coding properties. These markers are more conserved across taxa with less indel mutations and are much simpler to check for errors through amino-acid translation. Lack of recombination and uniparental inheritance of mitochondrial markers are added advantages [55]. The relatively high mitochondrial copy numbers also enable ease of amplification. The *cox*2 genetic marker, with its moderately high interspecific divergences within generic level and non-existent intraspecific variation, has demonstrated relatively successful results in terms of species identification in this study and is thus regarded as a potential mitochondrial DNA barcode for *Kappaphycus* and *Eucheuma*. Large scale application of this molecular marker is expected to bring about advancements mainly in the field of agriculture, taxonomy and biomonitoring. However, considering the already widely utilized *cox*2-3 spacer exhibited almost similar barcoding traits as that of the mitochondrial *cox*2 marker, continual implementation is recommended, in spite of its non-coding nature. The implementation of the *cox*2 molecular marker across taxa may pose a problem as well since the full potential of the *cox*2 marker has yet to be tested in other rhodophytes. Additionally, the massively abundant and readily available *cox*1 sequences within the GenBank (although not extensively tested on *Kappaphycus* and *Eucheuma* at that time, *cox*1 has been proposed as the potential universal DNA barcode for red algae) is apparently more practical and economical to work with, despite the relatively higher intraspecific variations which may reduce the accuracy for species identification [10], [18], [19], [46], [47], [48], [49]. Based on the results of this study, the authors have come to a consensus that the mitochondrial *cox*2-3 spacer was in overall the best potential DNA barcode for *Kappaphycus* and *Eucheuma*; whereas the combined mitochondrial-encoded *cox*1 and the plastid-encoded *rbc*L markers serve better as DNA barcodes encompassing the entire rhodophyte taxa.

The robustness and efficiency of DNA barcoding tends to increase with increased reference sequences and taxonomic scrutiny [1], [6], [44], [50]. This includes genetically distinct individuals within a species' range to account for molecular markers with high variability such as the *cox*1 [33]. Although this would greatly increase GenBank data and hence lead to an inevitable increment in terms of computational demand, larger and properly annotated datasets would expedite phylogeography, evolutionary biology, biodiversity or population genetic studies in the future.

### Large Dataset Assessment

An additional 46 GenBank *cox*2-3 spacer sequences of *Kappaphycus* and *Eucheuma* were compiled to simulate the effectiveness of a molecular marker in large, empirical datasets. Certain specimens of unknown or uncertain identity i.e. *Kappaphycus cottonii, Eucheuma isiforme* etc. were not used to avoid data confounding. Morphological plasticity of these seaweeds has rendered species identification and description challenging, even to seasoned taxonomists. As of now, distinctive morphological characters are still undiscovered despite ample of DNA evidence supporting the possible existence of new, or perhaps cryptic species. This has led to the amplification of Operational Taxonomic Units (OTU) in this context; representing genotypic diversity possibly overlooked via conventional morphological traits.

Distance-based results on the *cox*2-3 spacer dataset have shown that the incorporation of more sequences decreases the “barcoding gap” (when not overlapping), and to the extent of forming overlaps between inter- and intraspecific divergence. When *Kappaphycus* and *Eucheuma* species were regarded as non-OTUs, the “barcoding gap” for the entire length of *cox*2-3 spacer genetic marker decreased from 0.27% to 0%. When under OTU assortment, the intra- and interspecific genetic divergences formed an overlap of 0.29%. These observations were not surprising considering the larger sample size would eventually lead to higher occurrence of specimens with varying genetic composition [6], [45]. This would undoubtedly affect the identification accuracy of DNA barcoding. BCM results saw a slight dip from 93.1% to 90.7% when samples were regarded as OTUs. Similar patterns were observed for tree-based DNA identification under Meier's (2006) [33] criteria, where identification success reduced from 96.6% to 95.9% for non-OTUs, caused mainly by ambiguous sequences. These “singleton” sequences or query sequences sister to known species can be avoided with increased data and taxonomic rectification or reformation.

The distance-based *All Species Barcodes* (ASB) assessment, being relatively stricter compared to BM and BCM; has reflected our poor taxonomic comprehension on *Kappaphycus* and *Eucheuma*, in which at our current state (represented by non-OTU species assortment); a mere 61.3% of queries could be identified correctly using DNA barcoding. This figure is probably overestimated considering the large amount of *Kappaphycus* and *Eucheuma* yet to be discovered and described. Misidentifications under non-OTU conditions were caused by the African (KA2) and Hawaiian (KA3) *K. alvarezii* specimens, where the latter remained unresolved using the *cox*2-3 spacer and *cox*1 (results not shown) molecular markers. Still, combined *cox*2-3 spacer and RuBisCO spacer data has shown with moderate support that the KA3 specimens were more closely related to *K. alvarezii* than *K. striatum* [28]. This implies the possible limitation in resolving power of individual markers at intraspecific levels although the *rbc*L and *cox*2 molecular markers are yet to be tested. On the other hand, ASB has returned relatively higher identification successes (90.7%) when *Kappaphycus* and *Eucheuma* species were regarded as OTUs, thus providing invaluable insights into the possible taxonomic structure of these rhodophytes in the future.

### Phylogenetic Interpretation

The inclusion of GenBank sequences did not cause significant alterations to the phylogenetic tree topology (Figure 2 and Figure 5), apart from the additional clade KA3 composed of Hawaiian *Kappaphycus alvarezii* and clade EP consisting of *Eucheuma platycladum*. The monophyly of both *Kappaphycus* (NJ-BS = 100%; ML-BS = 99%; MP-BS = 100%; BI-PP = 1.00) and *Eucheuma* (NJ-BS = 100%; ML-BS = 78%; MP-BS = 100%; BI-PP = 0.92) was moderate to highly supported. Taxonomic statuses of specimens excluded in this study i.e. *Kappaphycus cottonii*, *Kappaphycus procrusteanus*, *Betaphycus philippinensis* and *Eucheuma isiforme* remained unaddressed at this time. Solid inferences can only be made when type specimens are sequenced, or when more reference sequences are available.

### Kappaphycus

Similar patterns of biodiversity as to that earlier reported were observed [23], [28], [29]. *K. alvarezii* KA1, being the most widely distributed genotype, was reported throughout Southeast Asia, Africa, Columbia, Panama as well as Hawaii. *Cox*2-3 spacer genealogy suggested that the African and Hawaiian strains of *K. alvarezii* KA1 may possibly be introduced strains, presumably traceable back to the Philippines. This is not surprising as there were efforts to introduce foreign, good strains of *Kappaphycus* for cultivation in the past [23], [28], [56]. Phylogenetic trees generated using the *cox*1, *cox*2 and *rbc*L DNA markers (Figure 2) have collectively displayed similar topologies constituted of similar specimens for clade KA1. This supports the report by Tan and co-workers (2012) [29] that local varieties of cultivated *K. alvarezii* do not differ genetically despite the distinctive morphologies, at least for Malaysian specimens. *K. alvarezii* KA2 and KA3 represent genotypes unique to Africa and Hawaii respectively. The taxonomic position of the Hawaiian *K.* “*alvarezii*” specimens (KA3) remained unresolved using the *cox*1 (results not shown) and *cox*2-3 spacer marker, where the Hawaiian samples occur as a polytomy to both *K. alvarezii* and *K. striatum*. Although GenBank sequences are limited, combined analysis using both the *cox*1 and *cox*2-3 spacer suggested that clade KA3 is sister to that of *K. alvarezii* and *K. striatum* (Results not shown). This contradicts the phylogenetic results based on the combined *cox*2-3 spacer and RuBiSCO spacer dataset, where the Hawaiian *Kappaphycus* specimens were inferred to be sister to *K. alvarezii* (KA1 and KA2 in this context) with moderate support (MP-BS = 70%) [28]. Application of the mitochondrial *cox*2 and plastid *rbc*L marker may better elucidate the current confusion associated with clade KA3. As earlier reported, two genotypes were observed for *Kappaphycus striatum* [28], [29], denoted here as clades KS1 and KS2. No distinctive differences in terms of gross morphology were identified between these two genotypes as of now and it is unsure whether cryptic species may apply in this situation. However, it appears that cultivated *K. striatum* mostly reside within clade KS1 and are largely common in Southeast Asia, although a recent study has also reported its occurrence in Uroa, Tanzania as a result of strain introduction [32]. *Kappaphycus* sp. “*Aring-aring*” from Malaysia was earlier shown to be phenotypically and genotypically different from *K. alvarezii* and *K. striatum* [29]; comparative studies against type specimens, particularly that of *Kappaphycus cottonii* (Weber-van Bosse) Doty ex P.C.Silva are currently underway in order to determine its validity as a new species.

### Eucheuma

Eucheumatoids are relatively poorly studied compared to *Kappaphycus*, possibly due to identification difficulties as well as lower economic value. *Eucheuma denticulatum*, being the more popularly cultivated species, were clustered into three genotypically distinct subclades. Subclade ED1 represents specimens from Southeast Asia and Hawaii, subclade ED2 from Southeast Asia, Hawaii and Tanzania [28] whereas samples within ED3 were exclusively from Africa. Despite coexisting in the South China and Celebes seas, Southeast Asian *Eucheuma denticulatum* ED1 and ED2 does not share similar morphological characteristics. This was shown by Ganzon-Fortes and co-workers [57], demonstrating the differences between the “*Endong*/*Spaghetti*” variety (ED2) from that of the usual *Spinosum* variety (ED1) of *Eucheuma denticulatum*. The “*Endong*” variety, thence named *E. denticulatum* (Burman) Collins & Hervey var. *endong* Trono & Ganzon-Fortes var. nov. exhibited smooth, slender terete axes with whorls of determinate branchlets at predictable intervals [57]. Recent samplings in Malaysia have also revealed that the local *Eucheuma denticulatum* “*Cacing*” variety fits the morphological and biochemical descriptions of the “*Endong*” variety, and its genotypic affinity also supported by *cox*1 and *rbc*L molecular data (data not shown). The apparent and distinctive morphological characters of the “*Endong*” variety does not fit the original descriptions for *E. denticulatum*, thus suggesting that it may be a new species instead of a rare variety. With reference to Figure 2, this was shown to be potentially true using the full length *cox*1 genetic marker, where the monophyly of clades ED1 and ED2 were highly supported. Relatively lower nodal supports were displayed by the *cox*2-3 spacer and *cox*2 DNA markers, followed lastly by the *rbc*L marker which fails to clearly indicate monophyly of ED1 and ED2. These patterns are reflective of the genetic variability of each molecular marker and suggest that clades ED1 and ED2 are probably undergoing divergence or have recently diverged. *E. denticulatum* ED3 was inferred to share a common ancestry with *E. denticulatum* ED1 and ED2, and are to date only reported in Africa. Considering the significantly different morphologies reported for ED2, it would be interesting to relook into the detailed anatomy of the African *E. denticulatum*.

## Supporting Information

Figure S1 **Neighbor-Joining** (**NJ**) **tree based on the** ***cox*** **2-3 spacer marker.** Numeric values at nodes indicate NJ bootstrap supports. *Large dataset assessment*: blue lines indicate Operational Taxonomic Units (OTU), whereas red lines represent non-OTU clusters. The *Solieria* outgroup was omitted from the analysis to enable implementation of tree-based identification criteria.
(TIF)

Figure S2 **Maximum Parsimony** (**MP**) **phylogenetic tree based on the** ***cox*** **2-3 spacer.** Number at nodes indicates MP bootstrap supports. *Large dataset assessment*: blue lines indicate Operational Taxonomic Units (OTU), whereas red lines represent non-OTU clusters.
(TIF)

Figure S3 **Bayesian** (**BI**) **phylogenetic tree based on the** ***cox*** **2-3 spacer DNA marker.** Number at nodes indicates BI posterior probabilities. *Large dataset assessment*: blue lines indicate Operational Taxonomic Units (OTU), whereas red lines represent non-OTU clusters.
(TIF)
