# Supplementary figures and images for: Assessment of Four Molecular Markers as Potential DNA Barcodes for Red Algae Kappaphycus Doty and Eucheuma J. Agardh (Solieriaceae, Rhodophyta)
Source: PLoS One. 2012 Dec 20;7(12):e52905. doi: 10.1371/journal.pone.0052905 (PMC3527623; doi:10.1371/journal.pone.0052905)

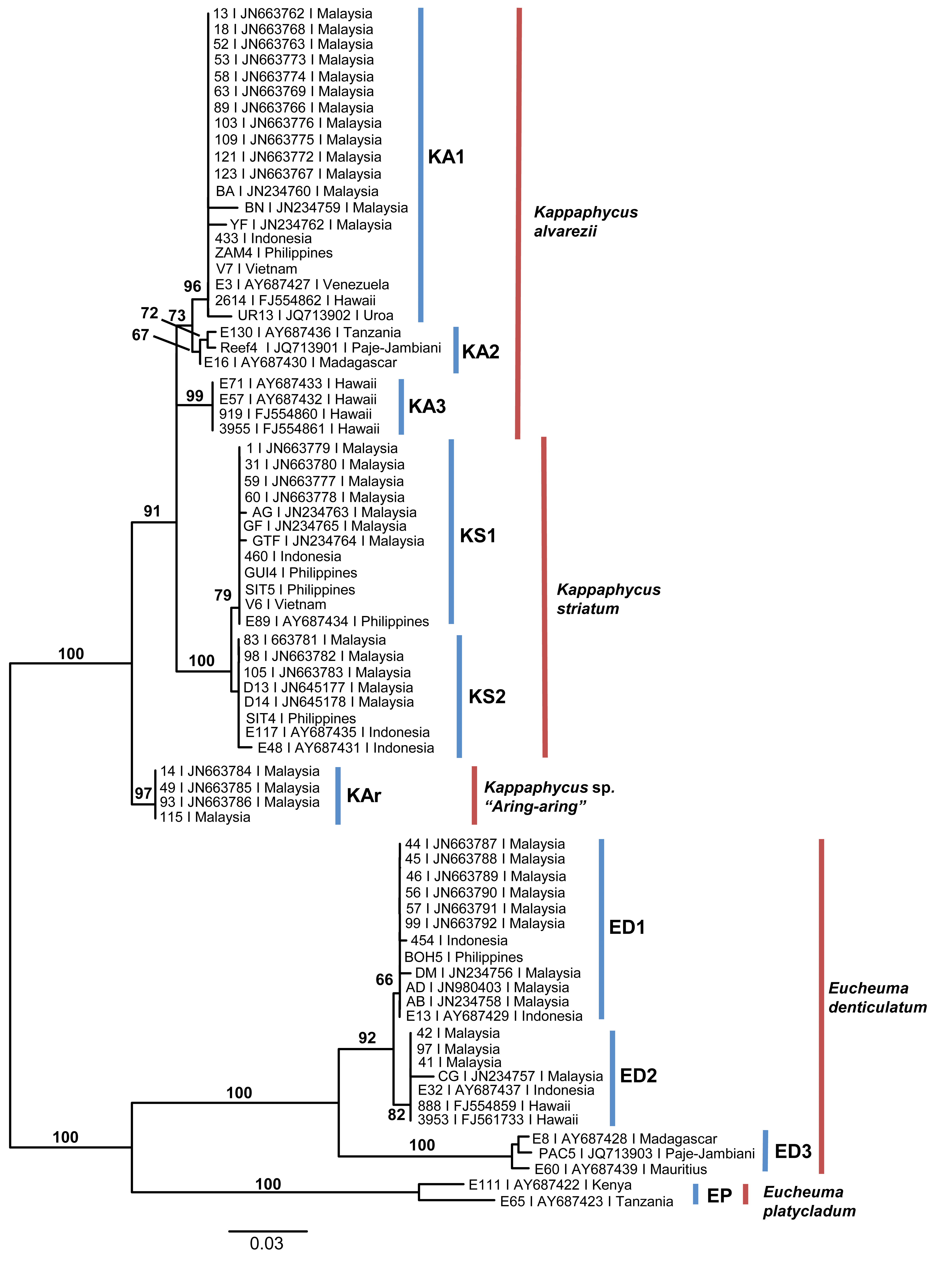

Supplement: Figure S1 — Neighbor-Joining (NJ) tree based on the cox 2-3 spacer marker. Numeric values at nodes indicate NJ bootstrap supports. Large dataset assessment: blue lines indicate Operational Taxonomic Units (OTU), whereas red lines represent non-OTU clusters. The Solieria outgroup was omitted from the analysis to enable implementation of tree-based identification criteria. (TIF) [file pone.0052905.s001.tif]

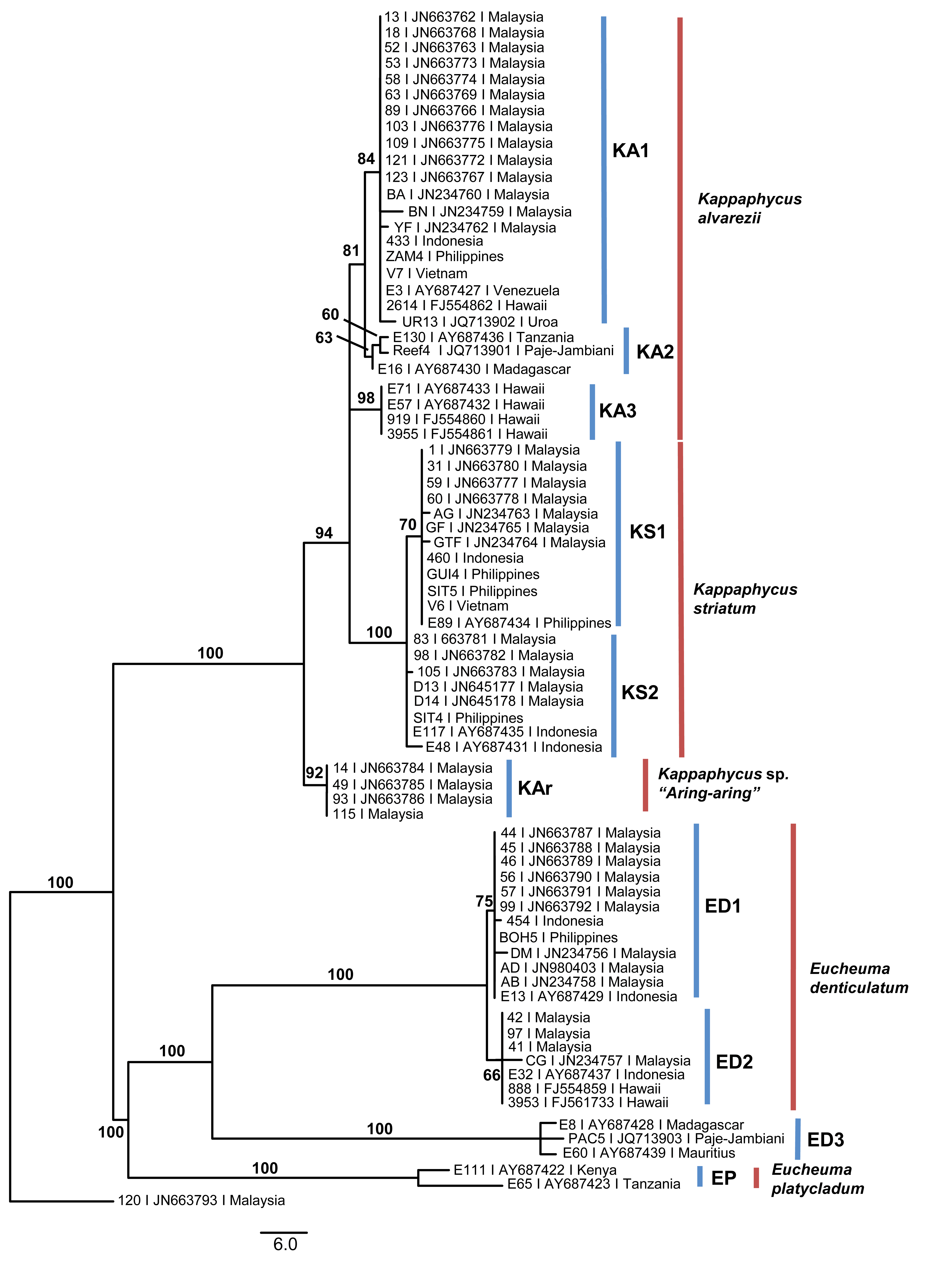

Supplement: Figure S2 — Maximum Parsimony (MP) phylogenetic tree based on the cox 2-3 spacer. Number at nodes indicates MP bootstrap supports. Large dataset assessment: blue lines indicate Operational Taxonomic Units (OTU), whereas red lines represent non-OTU clusters. (TIF) [file pone.0052905.s002.tif]

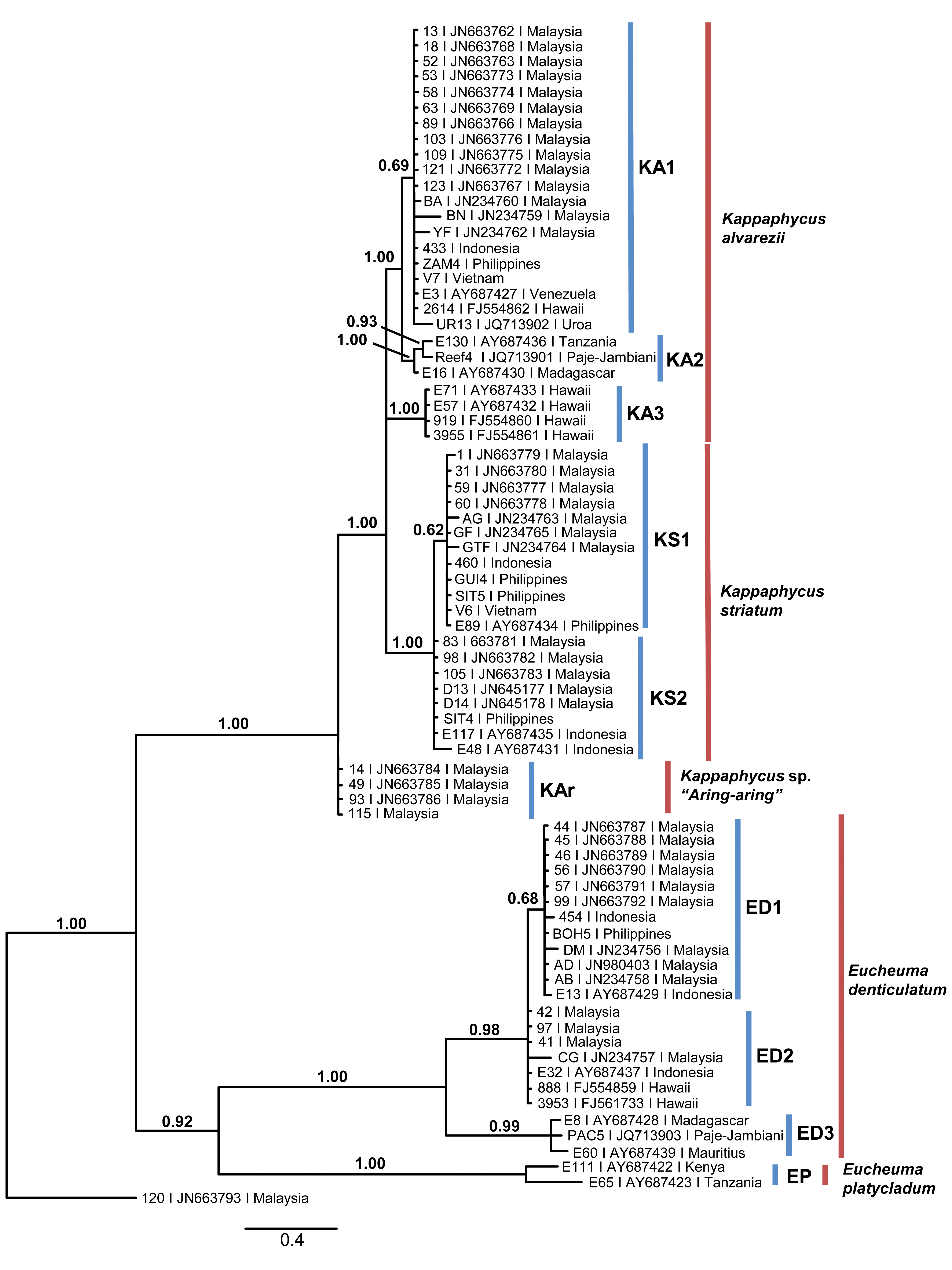

Supplement: Figure S3 — Bayesian (BI) phylogenetic tree based on the cox 2-3 spacer DNA marker. Number at nodes indicates BI posterior probabilities. Large dataset assessment: blue lines indicate Operational Taxonomic Units (OTU), whereas red lines represent non-OTU clusters. (TIF) [file pone.0052905.s003.tif]
